# A Severe Form of Mpox Infection and the Current Epidemiological Status in Romania

**DOI:** 10.3390/microorganisms13081814

**Published:** 2025-08-03

**Authors:** Anca Ruxandra Negru, David Valentin Mangaloiu, Ovidiu Vlaicu, Alexandra Cornovac, Violeta Molagic, Irina Duport-Dodot, Cătălin Tilișcan, Laurențiu Stratan, Adrian Marinescu, Lia Cavaropol, Mihaela Nicoleta Bercea, Andreea Marilena Păuna, Daniela Pițigoi, Victoria Aramă, Sorin-Stefan Aramă

**Affiliations:** 1 Faculty of Medicine, “Carol Davila” University of Medicine and Pharmacy, 37 Dionisie Lupu Street, 020021 Bucharest, Romania; anca1808@yahoo.com (A.R.N.); violeta_molagic@yahoo.com (V.M.); irina_duport@yahoo.com (I.D.-D.); catalin.tiliscan@gmail.com (C.T.); laurentiumstratan@gmail.com (L.S.); dramarinescu@yahoo.com (A.M.); andreea.pauna@umfcd.ro (A.M.P.); daniela.pitigoi@umfcd.ro (D.P.); or dr.arama@mateibals.ro (S.-S.A.); 2 National Institute for Infectious Diseases “Prof. Dr. Matei Balş”, 020021 Bucharest, Romania; vlaicu.ovidiu@yahoo.com (O.V.); alexandra.cornovac@rez.umfcd.ro (A.C.); liacavaropol@yahoo.com (L.C.); mihaela-nicoleta.bercea@rez.umfcd.ro (M.N.B.); 3 Medico-Military Institute, 020021 Bucharest, Romania

**Keywords:** Mpox epidemiology, Mpox relative viral load, necrotic skin lesions, AIDS, tuberculosis, pneumocystis pneumonia

## Abstract

Mpox has become a significant health concern since the global outbreak that began in 2022. The aim of this study is to present the epidemiological situation of Mpox in Romania during 2022–2023 and to describe a severe case of Mpox in a patient who survived despite multiple co-pathologies. Forty-seven confirmed cases were reported at the national level, all in men, in 2022. The median age was 33 years. Twenty-six cases involved men who have sex with men (MSM), and twenty-three tested positive for HIV. We also describe a severe case involving a 34-year-old bisexual male with newly diagnosed AIDS who developed severe Mpox with persistent necrotic skin lesions, respiratory involvement, and multiple opportunistic infections: tuberculosis, pneumocystis pneumonia, syphilis, and oral candidiasis. The patient presented with fever, night sweats, weight loss, and dyspnea, with a single ulcerative facial lesion that later disseminated. Mpox infection was confirmed through PCR from skin lesion, serum, saliva, urine, rectal, nasal, and pharyngeal swab samples, with high viral loads persisting despite prolonged Tecovirimat therapy. The patient developed immune reconstitution inflammatory syndrome following the initiation of antiretroviral therapy. This case emphasizes the challenges of treating Mpox in immunocompromised patients.

## 1. Introduction

Mpox infection, formerly known as monkeypox, is an infectious disease caused by the monkeypox virus (MPXV). Between 2022 and 2023, a global outbreak of MPXV was documented, with a disproportionate impact observed among men who have sex with men (MSM) [1].

MPXV was isolated for the first time in Denmark, between 1950 and 1960, from a colony of laboratory monkeys; a few years later, the virus was identified as a cause of disease in humans. After the cessation of smallpox immunization, which also protects against Mpox, the incidence of Mpox cases increased, especially in Central and East Africa. The first outbreak in the Western Hemisphere was registered in 2003 in the United States. After that, sporadic cases were reported in several non-endemic countries, mostly related to traveling from Africa [1,2].

A global outbreak of Mpox began in May 2022 and continues to this day, since in recent months the number of cases has been increasing in the Democratic Republic of Congo. On the 23rd of July 2022, after the emergence of many Mpox cases reported in previously non-endemic countries worldwide, the World Health Organization (WHO) declared a public health emergency of international concern [2,3]. The first cases of Mpox in this outbreak were identified in May 2022 in the United Kingdom and were not associated with recent travel in endemic areas. Meanwhile, more non-travel-related cases were reported in other European countries [4].

MPXV is an enveloped double-stranded DNA virus from the genus Orthopoxvirus of the Poxviridae family, along with the variola, cowpox, and vaccinia viruses. Currently, there are two known clades of MPXV, clade I and clade II, which have two subclades: a and b. Clade IIb is responsible for the outbreak that began in 2022. Small animals such as squirrels and monkeys are susceptible to MPXV and may be natural reservoirs for this virus [5]. Different strains of MPXV exhibit varying mortality rates, with clade I associated with higher case fatality ratios compared to clade II. A systematic review by Bunge et al. reported an overall case fatality rate of 8.7% across all countries [6,7,8]. Recent data indicate that Mpox has a variable case fatality rate, with historical estimates ranging from 1% to 11% and significantly higher mortality associated with the Central African (Congo basin) clade (up to 10.6%) compared to the West African clade (approximately 3.6%) [6].

MPXV is transmitted mainly from person to person through close contact (skin-to-skin, mouth-to-mouth, or mouth-to-skin contact) with an infected person. Other ways of transmission include contact with contaminated objects such as clothes or needle injuries. In utero transmission of MPXV may cause loss of the pregnancy, stillbirth, or other complications in both mother and newborn. Transmission from animals to humans occurs from infected animals through bites or scratches or during hunting, skinning, or cooking [1,9]. The incubation period ranges from 4 to 21 days. Systemic symptoms that occur before the rash, and last 1 to 5 days, are associated with the viremic phase of the disease and include fever, myalgia, fatigue, sore throat, and headache [4]. The characteristic rash progresses through several stages, from macules to papules, vesicles, and pseudo-pustules that crust over after several days. These lesions can affect any region of the skin. Moreover, oral, anal, and genital mucosae may also be affected [10]. Immunocompromised patients, especially patients with advanced HIV infection, are at risk of severe disease characterized by necrotizing skin lesions, pulmonary and gastrointestinal involvement, bacterial infections, and overall high mortality rates [11].

Clinical management of patients diagnosed with Mpox involves supportive care with or without antiviral treatment. While most immunocompetent patients develop a mild form of the disease followed by recovery without medical treatment, immunocompromised persons may develop a severe form that requires antiviral therapy. Tecovirimat was the most frequently used antiviral agent during the 2022 outbreak. Alternatives to tecovirimat are Cidofovir and Brincidofovir. These molecules can also be prescribed together, especially in patients with immunocompromised conditions. To prevent disease spread, the vaccine can be administered to high-risk patients before or immediately after contact with an infected person [11].

Although the worldwide incidence of Mpox is decreasing, there are geographic regions where the number of diagnosed cases is rising [1,11]. Since January 2022, a total of 124,753 laboratory-confirmed Mpox cases and 272 deaths have been reported to the WHO across 128 member states from all six regions. The most affected countries worldwide since the outbreak include the United States (34,490 cases), Brazil (13,429), Spain (8481), France (4383), Colombia (4285), Mexico (4201), the United Kingdom (4166), Germany (4090), and Peru (3949). These numbers add up to 65% of all reported cases worldwide. Mpox remains a major health concern in Africa, with over 40.456 confirmed cases and 150 deaths reported to the WHO since January 2022. Limited diagnostic capacity and inconsistent surveillance systems in several countries likely contribute to underreported case numbers, affecting the accuracy of epidemiological assessments. Since the beginning of the outbreak, Mpox cases have been reported to the WHO from 30 African nations, with the majority in the past 12 months recorded in the Democratic Republic of the Congo (17,824 cases), Uganda (6479), and Burundi (3960) [12].

This study aims to illustrate the epidemiological status of Mpox in Romania and details a severe Mpox case in a patient who survived, despite having multiple comorbidities.

## 2. Materials and Methods

### 2.1. Participants and Samples

We performed a retrospective and descriptive study based on data available and published by the National Institute of Public Health of Romania (INSP) in 2022–2023 and on the data of Mpox patients confirmed in our hospital (Professor Dr. Matei Balș National Institute for Infectious Diseases (INBIMB), Bucharest) [13].

We used a series of epidemiological, clinical, and laboratory information from the following:

- Electronic Patients Records: Data were extracted from the digital system implemented at INBIMB for epidemiological and clinical analysis, for both suspected and confirmed cases. An Mpox case surveillance form was completed according to INSP guidelines and subsequently reported per Government Decision No. 657/2022 and Ministry of Health Order No. 1738/2022 [14]. Epidemiological, demographic, and clinical data were systematically recorded and processed using Excel software 2024, ensuring adherence to standardized Mpox case classification protocols.
- Clinical and laboratory analysis: Clinical data regarding symptoms and other potentially complicating infections (especially HIV, hepatitis B and C virus, and sexually transmitted infections—STIs) were collected at the patients’ first hospital presentation in the emergency unit. Furthermore, in patients with suspected or living with HIV-1 infection, HIV-1 viral load (VL) and CD4+ and CD8+ T cells in peripheral blood were quantified. Multiple samples were dynamically (approximately every 3 weeks for 5 months) collected from different sanctuaries (sera, skin lesion, saliva, urine, rectal, nasal, and pharyngeal swabs) from one Mpox-positive patient with HIV-1 in order to evaluate MPXV VL evolution. All clinical samples were collected in VTM (viral transport medium) tubes. Approximately 300 μL of VTM media was used for nucleic acid isolation using the QIAmp DSP Virus Kit (Qiagen, Germany) according to the manufacturer’s instructions. MPXV genome detection was performed with the monkeypox virus genesig^®^ Advanced Kit (Primer design, UK) according to the manufacturer’s instructions. The qPCR reactions were performed on the CFX96 thermal cycler (Bio-Rad, USA).
- Whole-genome sequencing (WGS): In order to understand the evolution of MPXV intra-host diversity from patients whose samples were dynamically collected, we performed WGS analyzing mutational sites. The best cycle threshold (Ct) samples (Ct < 25) were selected from two different sanctuaries (zygomatic skin lesion and rectal swabs) to identify potential viral evolution. Approximately 250 ng of extracted nucleic acid was used for next-generation sequencing (NGS) by Illumina DNA Prep (Illumina, USA) following the manufacturer’s recommendations. The reads were polished, trimmed, and de novo-assembled using the shovill pipeline with SPADES 3.12.067 already implemented [15,16]. The resulting contigs were further used for a BLAST reference sequence search [17]. The contigs as well as the raw reads were mapped on the best hit reference sequence. The MPXV strains underwent reference mapping, with trimming in a 5-repeat iteration fashion and the addition of a Q30 quality factor threshold, all performed with Geneious Prime 2023.0.1. The resulting consensus sequences for each of the sequenced strains were further subject to mutational diversity analysis. The sequences were aligned using Mafft [18].

### 2.2. Ethical Considerations

All research procedures were conducted according to applicable ethical standards and regulations, including the Declaration of Helsinki. Written informed consent was obtained from all the participants hospitalized in our institute. The Ethics Committee of the INBIMB approved this study (C01375/07.02.2025).

## 3. Results

### 3.1. Epidemiological Situation in Romania

In Romania, 47 confirmed cases were reported in 2022. During 2023, an additional seven suspected cases were reported, but none were confirmed with Mpox [13].

All cases involved men aged 20 to 54 years (Table 1), with a median age of 33. Most cases were reported among residents of Bucharest [13].

Based on data provided by the patients, most cases were found in MSM patients (*n* = 26), followed by bisexual persons (*n* = 12) and heterosexual patients (*n* = 9).

Only 33 patients offered information about the number of sexual partners in the last 3 months in Romania and/or outside the country (Table 2).

In the assessment of patients’ history of STIs, the most frequently reported condition was HIV-1, diagnosed in 23 individuals, representing 44.7% of the study cohort. One patient reported a dual history of HIV-1 and syphilis, while another patient disclosed a prior diagnosis of Chlamydia Spp. Another case involved a history of syphilis. Additionally, one patient had a documented history of tuberculosis. A total of 26 patients (55.3%) required continuous hospitalization. Notably, one individual with multiple underlying comorbidities needed an extended hospital stay; the details of this case are discussed below. No fatalities were recorded in the analyzed patient population.

In total, 19 (40.4%) out of the 47 confirmed cases in Romania were investigated and treated in our hospital.

Out of them, twelve patients were people living with HIV. The CD4+ T-cell count ranged from 53 to 797 cells/mm^3^, with a median of 551.15 cells/mm^3^. Eleven patients were on antiretroviral therapy at the time of Mpox diagnosis, of whom eight patients had undetectable HIV-1 VL. One patient was diagnosed with both HIV-1 and Mpox simultaneously. The most commonly reported signs and symptoms included skin and/or mucosal lesions, fever, sweating, myalgia, fatigue, dysphagia, and headache. Only three patients required hospitalization.

### 3.2. Case Description

**Figure 1:**
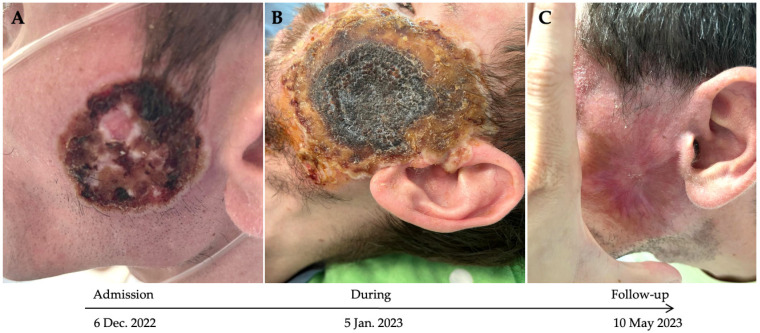
The evolution of an exemplary zygomatic skin lesion over the five months. (**A**) Zygomatic skin ulceration upon admission; (**B**) negative evolution during hospitalization; (**C**) zygomatic skin reconstruction at follow up.

We present the case of a 34-year-old bisexual male with no prior medical history who was admitted to INBIMB on 6 December 2022, complaining of fever, chills, profuse night sweating, weight loss of about 20 kg over the past three months, dry cough, and dyspnea. The onset of these symptoms was three months before presentation and gradually worsened over time. Prior to admission, the patient was evaluated at various other medical facilities and diagnosed with community-acquired pneumonia, but without clinical criteria for hospitalization. He was treated with multiple broad-spectrum antibiotics; however, his condition progressively declined. He developed acute respiratory failure and underwent a pulmonary CT scan in a private healthcare setting, which revealed bilateral cylindrical bronchiectasis and diffuse ground-glass opacities in the left inferior, right inferior, and middle lobes. Despite these findings, the patient refused admission. In addition to the respiratory symptoms, he had a solitary skin ulcer on the left zygomatic region measuring approximately 3 cm in diameter, with a black necrotic center, perilesional edema, and serous exudate. He reported that the ulcer appeared as a pustule three weeks prior to admission (Figure 1A).

Clinical examination revealed fever, dyspnea, high respiratory rate, and a resting blood oxygen level of 87%, which rose to 94–97% under oxygen therapy with a rate of 2 L/min. There were no crackles on pulmonary auscultation, but the vesicular murmur was diminished bilaterally. Oral cavity inspection revealed generally inflamed mucosa with white deposits. Anal examination showed external hemorrhoids, condylomas, and lesions compatible with genital herpes. Given these symptoms, the patient was admitted to a private ward for further clinical and laboratory investigations. The blood tests revealed AIDS (HIV—1 viral load, 2,750,000 copies/mL; CD4+ T cell count—53 cells/mm^3^; and CD8+ T cell count—517 cell/mm^3^) and latent syphilis (VDRL-positive and TPHA-positive, without chancre or other clinical symptoms to suggest acute infection). The cultures made from oral lesions were positive for *Candida albicans*. All blood culture tests were negative for both bacteria and fungi. Bronchoscopy was performed for diagnostic purposes and pulmonary tuberculosis was confirmed through PCR from bronchoalveolar lavage (no Rifampicin resistance mutations were present). Pneumocystosis was also confirmed through Grocott stain. The histopathologic examination from the skin lesion biopsy of the zygomatic area revealed unspecific dermo-epidermal ulceration.

Anti-tuberculous treatment was started with Isoniazid 2000 mg daily, Rifampicin 450 mg daily, Pyrazinamide 1000 mg daily, and Ethambutol 800 mg daily, along with Cotrimoxazole (20 mg TMP/kg per day) and adjunctive Prednisone in tapered doses for 21 days for pneumocystosis treatment. For oropharyngeal candidiasis, intravenous Fluconazole 400 mg/day was administered for 14 days. The patient also received three doses of Benzathine penicillin G—a total of 2,400,000 IU (day 0, day 7, and day 14)—against latent syphilis.

Initially, the clinical evolution was favorable, with remission of fever and respiratory symptoms, but the skin lesion continued to increase in size, and the perilesional edema worsened (Figure 1B). Three weeks after admission, the fever reappeared, and a new nodular lesion appeared on the left popliteal area, followed by several other lesions that appeared on his lips, tongue, leg, elbow, and nasal mucosa. The patient also complained about digestive symptoms (abdominal pain, mild diarrhea, and rectal pain).

All blood cultures and the laboratory results from stool samples were negative. The patient refused the colonoscopy.

Due to the epidemiological data obtained from the patient, who denied any sexual intercourse in the last 2 years, and the nonspecific aspect of the primary skin lesion, other possible etiologies were investigated: skin biopsy and swabs for bacterial, mycobacterial, and fungal cultures were obtained, all of them with nonspecific or negative results. The suspicion of Mpox infection was raised three weeks after admission, when new lesions with characteristic aspects of Mpox rash appeared (red spots that transformed into vesicles and ulcerations).

**Figure 2:**
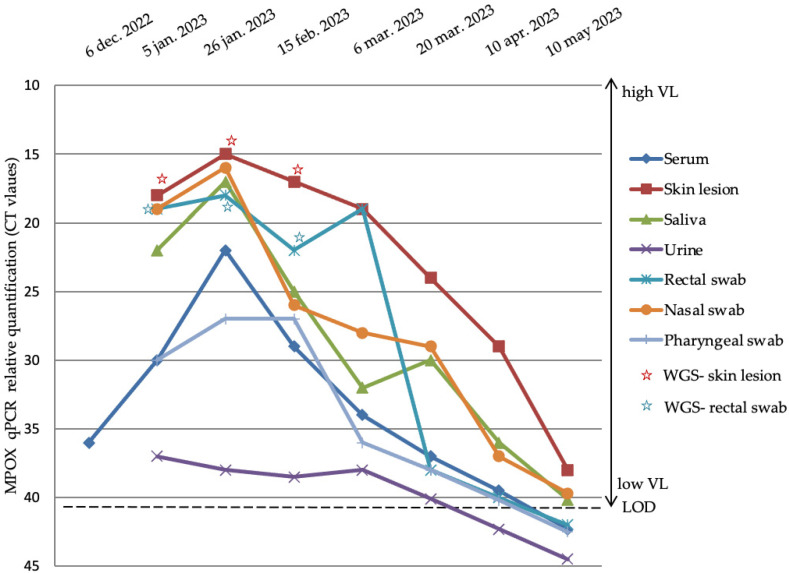
Dynamics of MPXV relative quantification from different samples. For five months, samples from different sanctuaries (serum, zygomatic skin lesion, saliva, urine, rectal, nasal, and pharyngean swabs) were collected for MPXV VL monitoring. Samples for WGS were collected from two different sanctuaries (serum and rectal swabs). LOD—limit of detection; WGS—whole-genome sequencing.

At the beginning of January 2023, samples from different sanctuaries were collected for suspected Mpox infection, although the patient claimed not to have had sexual intercourse in the last 2 years. The patient’s negative evolution combined with the worsening of the zygomatic ulceration led to a more thorough epidemiological investigation. Laboratory analyses showed the presence of MPXV in all samples (serum, zygomatic skin lesion, saliva, urine, rectal, nasal, and pharyngeal swabs), with notable differences in VLs between samples. The initial epidemiological assessment did not suggest an Mpox infection (Figure 2). Analyzing the serum sample used for HIV diagnosis in December 2022 allowed us to confirm MXPV infection retrospectively.

Antiviral treatment with Tecovirimat 600 mg bid was initiated; however the clinical evolution was not favorable, as the patient developed daily fever spikes, new skin lesions appeared, and the zygomatic lesion progressed.

Viral shedding in the serum showed transient viremia with rapid control, which was controlled under Tecovirimat therapy. Urine samples maintained the lowest viral loads, suggesting limited urinary involvement. We observed that skin lesions and the oropharynx served as primary infection sites, displaying the highest viral loads and prolonged persistence. These findings highlight the varying kinetics of viral clearance across body sites.

Regarding the intra-host genetic diversity, using the built-in tools available on the GISAID platform, we performed a mutation analysis at three different time points (5 January 2023, 26 January 2023, and 15 February 2023) in two different sites: the zygomatic skin lesion and rectum (Figure 2). The low relative VL of serum and rectal swab samples (Ct > 25) did not allow whole-genome sequencing (WGS) to be performed subsequently.

Overall, six MPXV WGSs were obtained from this patient. When comparing the two viral reservoirs, five mutations were found: E11L (V619I), F4l (E253K), G8L (D212N), G10R (R194H), and Q1L (M1071I) genes. Phylogenetic analysis was performed to classify the MPXV strains, and the results have been previously described [19]. The sequences obtained from this patient formed a single well-supported cluster, suggesting that there was no reinfection.

**Figure 3:**
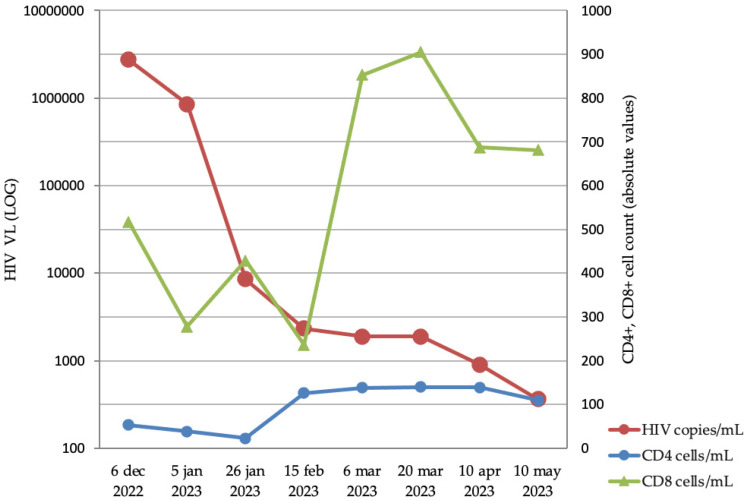
Summary of the evolution of both the HIV infection (relative VL normalized to the maximum measured levels) and the immunological reconstitution of the patient (in terms of rising CD4+ and CD8+ T cell count absolute values) following the start of ART at the end of 2022.

After three months of hospitalization, most skin lesions resolved without any scar; however, a few developed hyperpigmented scars and the facial lesion developed a mild retractile scar (Figure 1C). The patient followed the treatment with Tecovirimat for three months, after which he decided to discontinue it. He underwent anti-tuberculous therapy for 10 months and the antiretroviral treatment was switched to TAF/FTC/BIC as soon as the Rifampin-containing treatments were over. HIV-1 VLs and CD4+/CD8+ T cell counts were monitored simultaneously for five months (Figure 3). During the patient’s hospitalization and two months after discharge, the patient followed the antiviral treatment regimen. At follow-up visits (every 6 months), unfortunately, the patient was not adherent to the antiretroviral treatment, as the HIV-1 viral load was never undetectable. The highest value of CD4+ T cell count was 139 cells/mm^3^, and the nadir CD4+ T cell count was 38 cells/mm^3^.

## 4. Discussion

This study provides both an epidemiological overview of Mpox in Romania during the 2022–2023 period and an in-depth clinical analysis of a severe case occurring in the context of newly diagnosed AIDS and multiple co-infections. While the national burden of Mpox remained relatively low, the presented case highlights the potential for severe and prolonged disease in immunocompromised hosts, particularly those with advanced HIV infection.

### 4.1. Epidemiological Situation of Mpox Infection Worldwide and in Romania 

The recent global outbreak of Mpox represents a major shift in the epidemiology of this disease, which was previously considered an endemic threat limited to Central and West Africa. The rapid spread of the virus to countries outside of its typical regions of endemicity has highlighted the risk of zoonotic diseases as a cause of global health crises. The Mpox outbreak during this period is notable for both its geographic spread and the population affected, with a significant number of cases reported among MSM [4].

Worldwide, the outbreak impacted 128 countries and resulted in a significant number of cases, with a cumulative total exceeding 124,000 confirmed cases by the end of 2024. Although the vast majority of reported cases were mild, this outbreak had a significant number of severe cases, particularly among immunocompromised individuals, such as those living with HIV-1/AIDS, which considerably complicates the clinical management. The high prevalence of severe outcomes in immunocompromised patients is a stark reminder of the vulnerability of these populations, especially those with poorly controlled HIV-1 infection [3,11].

The first case was confirmed in Romania in a 26-year-old male patient with HIV who developed a mild form of the disease and was successfully treated with symptomatic and topical treatment at Victor Babeș Hospital for Infectious and Tropical Diseases in Bucharest [20]. In this study, we present the results of the national Mpox surveillance and the clinical data available for 47 patients diagnosed with Mpox in specialized hospitals for infectious diseases from Romania. Confirmed cases reported nationwide in 2022 occurred exclusively in male individuals. Twenty-six cases involved men who have sex with men (MSM) individuals, and twenty-three tested positive for HIV. The disease was diagnosed especially in young adults (20–39 years old), the same age group being the most affected at the European level [12,13]. No deaths occurred in the studied group.

### 4.2. Mpox Infection in Immunocompromised Patients

In our study we describe the case of a patient with multiple underlying comorbidities who necessitated an extended hospital stay. The case is particularly significant because, despite being severely immunocompromised and having multiple opportunistic infections, the patient survived a severe form of Mpox with extensive necrotic skin lesion and proctitis characterized by intense anorectal pain in association with a positive fecal occult blood test and pulmonary involvement with multiple bilateral pulmonary nodules. Given the patient’s condition, it is more likely that the pulmonary involvement was primarily caused by tuberculosis and pneumocystosis rather than Mpox itself, although in other studies, cases with pulmonary involvement presenting as nodules have been described [21]. This contrasts with the generally poor prognosis seen in other immunocompromised individuals with advanced HIV-1 infection. Numerous studies have concluded that severe complications were more common in people with a low CD4+ T cell count (under 100 cells/mm^3^). These severe cases are characterized by necrotizing skin lesions, lung involvement, and secondary infections and sepsis. Also, almost all deaths occurred in people with a low CD4+ T cell count and detectable viral load [22,23].

Along with HIV infection, other causes of immunodepression (malignancies and organ transplants) have been associated with severe forms of Mpox infection. A CDC study involving 57 patients hospitalized with severe Mpox infection revealed that 90% had CD4 counts below 200, with fewer than 10% receiving antiretroviral therapy. In total, 5% of the patients had undergone solid organ transplants, and 3.5% presented with hematologic malignancies. All subjects exhibited severe dermatological lesions, with the majority also experiencing mucosal complications. Moreover, Mpox infection disseminated systemically, involving the lungs (21% of cases), eyes (7%), and brain (7%). The mortality rate within the intensive care unit was 21%, with five of twelve fatalities directly attributable to Mpox [24].

Considering the progression of our patient’s Mpox infection—characterized by clinical deterioration, recurrence of fever, and the emergence of multiple skin lesions along with new pulmonary nodules detected on a CT scan approximately three weeks after initiating antiretroviral therapy (ART)—we suspect the occurrence of immune reconstitution inflammatory syndrome (IRIS) related to Mpox. This condition is noted to have a high mortality rate in most studies [5,10,25,26].

Several reports have noted that Mpox symptoms can worsen after the initiation of ART, which is typically considered a form of IRIS. However, some studies suggest that the degradation of Mpox infection in people with uncontrolled HIV-1 may be related to the immune response to the infection itself, rather than solely being attributed to IRIS [25,27].

### 4.3. Virological Evolution of Mpox Infection

In the described case, relative viral loads from skin lesions, saliva, rectal, nasal, and pharyngeal swabs and their dynamics over a period of approximately five months showed a gradual and asynchronous decline. Data were collected from multiple anatomical sites, providing a comprehensive perspective on viral kinetics and the dissemination of the infection. Over several months, a general trend of decreasing viral loads was present in most sites, although some, like skin, nasal, and rectal swabs, showed later peaks or more prolonged detection, despite Tecovirimat treatment.

Regarding etiological treatment, Tecovirimat is the most used antiviral drug worldwide. Despite receiving Tecovirimat, the Mpox VL remained positive for a long period of time. Unfortunately, we could not perform susceptibility tests. In other studies, the rate of resistance to Tecovirimat was low (<1%). Mutations in the gene that encodes the target protein of Tecovirimat (OP57 gene) might lead to Tecovirimat resistance. Our patient was at risk of developing resistance to Tecovirimat because prolonged disease evolution and selective pressure due to antiviral treatment, especially in immunocompromised hosts, can lead to the acquisition of resistance-conferring mutations [28,29].

The patient’s ability to survive despite extensive disease highlights the complexity of Mpox infections, suggesting that with appropriate care, outcomes may be more favorable than previously reported for similar cases.

This case emphasizes the critical need for early recognition and diagnosis of Mpox in patients with compromised immune systems. Clinicians should be vigilant for Mpox in any patient with relevant risk factors, especially those presenting with unusual skin lesions, respiratory symptoms, or constitutional signs such as fever and weight loss. Additionally, this case highlights the necessity for comprehensive care strategies that address the various aspects of patient management in the context of co-infections. In patients with Mpox, concurrent infections like tuberculosis, pneumocystis pneumonia, and syphilis may complicate the clinical course and treatment regimens must be tailored accordingly.

Several limitations of our study need to be highlighted. First, the limited number of cases analyzed, combined with the likelihood that some mild infections went undiagnosed, restricted the depth and generalizability of this study’s findings. Another limitation of our study was that, being based on patient-reported data, information on exposure and sexual behaviors was difficult to obtain, and data were often incomplete. Additionally, we did not have the capacity to detect the specific gene mutation associated with Tecovirimat resistance, which may have contributed to the extended presence of viral material across multiple anatomical sites.

## 5. Conclusions

This case underscores the importance of early recognition and comprehensive care for Mpox, especially among patients with significant comorbidities, and highlights the need for ongoing epidemiological surveillance in Romania. While most patients with Mpox infection recover with minimal intervention, individuals with advanced HIV-1 infection and other co-infections face a higher risk of severe disease. This case, which had a favorable outcome despite the patient’s significant immunodeficiency, emphasizes the potential for positive results when employing comprehensive, multidrug treatment strategies. It also offers valuable insights into managing Mpox in patients with complex clinical conditions while providing a comprehensive overview of the epidemiological and clinical characteristics of Mpox cases both at the national level and within our medical facility.

## Figures and Tables

**Table 1:** Demographic characteristics of confirmed Mpox cases, 2022 (*n* = 47).

Characteristics	Number of Cases
Male	47/47
**Age group**	
20–29 years	12/47
30–39 years	25/47
40–49 years	8/47
>50 years	2/47
**Residence**	
Bucharest (capital)	38/47
Other districts	9/47

**Table 2:** The number of sexual partners in the last 3 months in Romania and outside the country of Mpox cases.

Country	Number of Sexual Partners in the Last 3 Months	*n*
Romania and other countries	1 2 3 10	16/47 13/47 5/47 1/47
Not available data		*n* = 12/47

*n*—number.

## Data Availability

The data presented in this study are available on request from the corresponding author.
